# Examining the efficacy of intravenous administration of predatory bacteria in rats

**DOI:** 10.1038/s41598-017-02041-3

**Published:** 2017-05-12

**Authors:** Kenneth Shatzkes, Eric Singleton, Chi Tang, Michael Zuena, Sean Shukla, Shilpi Gupta, Sonal Dharani, Joseph Rinaggio, Daniel E. Kadouri, Nancy D. Connell

**Affiliations:** 1 Division of Infectious Disease, Department of Medicine, Rutgers New Jersey Medical School, Newark, NJ 07103 USA; 2 Department of Oral Biology, Rutgers School of Dental Medicine, Newark, NJ 07103 USA; 3 Department of Diagnostic Sciences, Rutgers School of Dental Medicine, Newark, NJ 07103 USA

## Abstract

The proteobacteria *Bdellovibrio bacteriovorus* and *Micavibrio aeruginosavorus* are obligate predators of Gram-negative bacteria, and have been proposed to be used to treat multidrug-resistant bacterial infections. The ability of predatory bacteria to reduce bacterial burden *in vivo* within the lungs of rats has been demonstrated, but it was unknown if predatory bacteria can attenuate systemic bacterial burden administered intravenously. In this study, we first assessed the safety of intravenous inoculation of predatory bacteria in rats. No rat morbidity or adverse histopathology of various organs due to predatory bacteria administration was observed. An increase in proinflammatory cytokines (TNFα and KC/GRO) was observed at two hours post-inoculation; however, cytokines returned to baseline levels by 18 hours. Furthermore, bacterial dissemination analysis demonstrated that predatory bacteria were efficiently cleared from the host by 20 days post-injection. To determine whether predatory bacteria could reduce bacterial burden *in vivo*, *Klebsiella pneumoniae* was injected into the tail veins of rats and followed with multiple doses of predatory bacteria over 16 or 24 hours. Predatory bacteria were unable to significantly reduce *K. pneumoniae* burden in the blood or prevent dissemination to other organs. The results suggest that predatory bacteria may not be effective for treatment of acute blood infections.

## Introduction

*Bdellovibrio bacteriovorus* and *Micavibrio aeruginosavorus* are Gram-negative proteobacteria that are obligate predators of other Gram-negative bacteria^1, 2^. *B. bacteriovorus* attach to and invade their prey by crossing the outer prey cell membrane and establishing themselves in the periplasmic space, forming a bdelloplast^3–5^. There, the growing *B. bacteriovorus* exhausts the contents of the prey cell before dividing by septation, lysing the bdelloplast, and then continuing to seek out more prey to invade. In contrast, the epibiotic predator *M. aeruginosavorus* does not invade bacterial cells, but rather attaches to the outer membrane and kills its prey from the outside in ‘vampire’ fashion^2, 6, 7^.

In response to the recent rise of multi-drug resistant (MDR) bacterial infections, the use of predatory bacteria has been proposed as a novel alternative therapy. Predatory bacteria are effective against many Gram-negative human pathogens *in vitro* ^8^, including biofilm-associated^9–11^ and MDR infections^12^, and as a further advantage, genetically-encoded predation resistance has yet to be confirmed^13^. Multiple studies have shown predatory bacteria to be non-toxic in a number of animal models such as mice, rats, chicks, guinea pigs, and rabbits^14–18^. A study performed in our laboratory was the first to demonstrate the ability of predatory bacteria to reduce bacterial burden *in vivo* within the lungs of rats^19^. Most recently, injections of predatory bacteria were shown to cooperate with host immune cells to treat *Shigella* infection in the hindbrains of zebrafish larvae, leading to increased zebrafish survival^20^. However, it is still unknown if predatory bacteria can reduce pathogen burden introduced into an animal through other modes of administration, including directly into the bloodstream. This is of interest not only as a potential alternative to treat MDR-infections, but also as a potential therapeutic for sepsis triggered by Gram-negative pathogens.

In this study, first, the safety of injecting predatory bacteria directly into the tail vein of rats was evaluated. Then, rats were intravenously inoculated with *Klebsiella pneumoniae* and treated with multiple doses of predatory bacteria to determine their ability to attenuate pathogen burden within the vasculature of an *in vivo* system. The work presented here further addresses concerns with the idea of developing predatory bacteria into a novel antimicrobial.

## Results

### Host morbidity and histopathology

To examine the long-term effect of intravenous inoculation of predatory bacteria on rat morbidity, we injected 1.3 × 10^9^ plaque forming units (PFU)/rat of *B. bacteriovorus* strain 109J or 1.3 × 10^8^ PFU/rat of *M. aeruginosavorus* strain ARL-13 into the tail veins of two groups of 24 Sprague-Dawley (SD) rats each. Another group of 24 rats was inoculated with the vehicle, phosphate buffered saline (PBS). At 10 days post-initial inoculation, twelve rats from each of the *B. bacteriovorus*-treated and *M. aeruginosavorus*-treated groups were re-injected with 1.8 × 10^9^ PFU/rat of *B. bacteriovorus* or 3.9 × 10^8^ PFU/rat of *M. aeruginosavorus*, respectively, to model a multiple bacteremic event. Twelve rats from the PBS-treated group were also re-injected with PBS. All animals were monitored for an additional 10 days (for 20 days total) for any signs of illness, infection, or discomfort. At 20 days post-initial injection, all rats that received either single or multiple injections with PBS or predatory bacteria were found to be visually healthy with no signs of illness or discomfort (Table 1).

**Table 1:** Numbers of rats showing visual signs of morbidity after tail vein injection with predatory bacteria

Treatment	No. of rats showing visual signs of morbidity at indicated time after inoculation/total no. of rats				
	2 h	4 h	18 h	20 d	20 d*
Control (PBS)	0/12	0/12	0/12	0/12	0/12
*B. bacteriovorus* 109 J	0/12	0/12	0/12	0/12	0/12
*M. aeruginosavorus* ARL-13	0/12	0/12	0/12	0/12	0/12
*K. pneumoniae* ^a^	0/7	0/7	1/7		

*Rats were inoculated a second time at 10 days post-infection to model a multiple bacteremia event. ^a^With animal well-being in mind, rats inoculated with *K*. *pneumoniae* were not kept past 18 h post-inoculation.

To examine the short-term effect of introducing predatory bacteria into the bloodstream on rat morbidity and histopathology, we injected 1.7 × 10^9^ PFU/rat of *B. bacteriovorus* 109J or 2.3 × 10^8^ PFU/rat of *M. aeruginosavorus* into the tail veins of two groups of 36 SD rats each. Another group of 36 rats were injected with the vehicle, PBS, while an additional 21 rats were injected with 1.8 × 10^8^ CFU/rat of *K. pneumoniae*, a known pathogen. Twelve animals from each group (seven for *K. pneumoniae*) were sacrificed at two, four, and 18 hours post-inoculation when blood was collected and organs harvested for further analysis.

**Figure 1:**
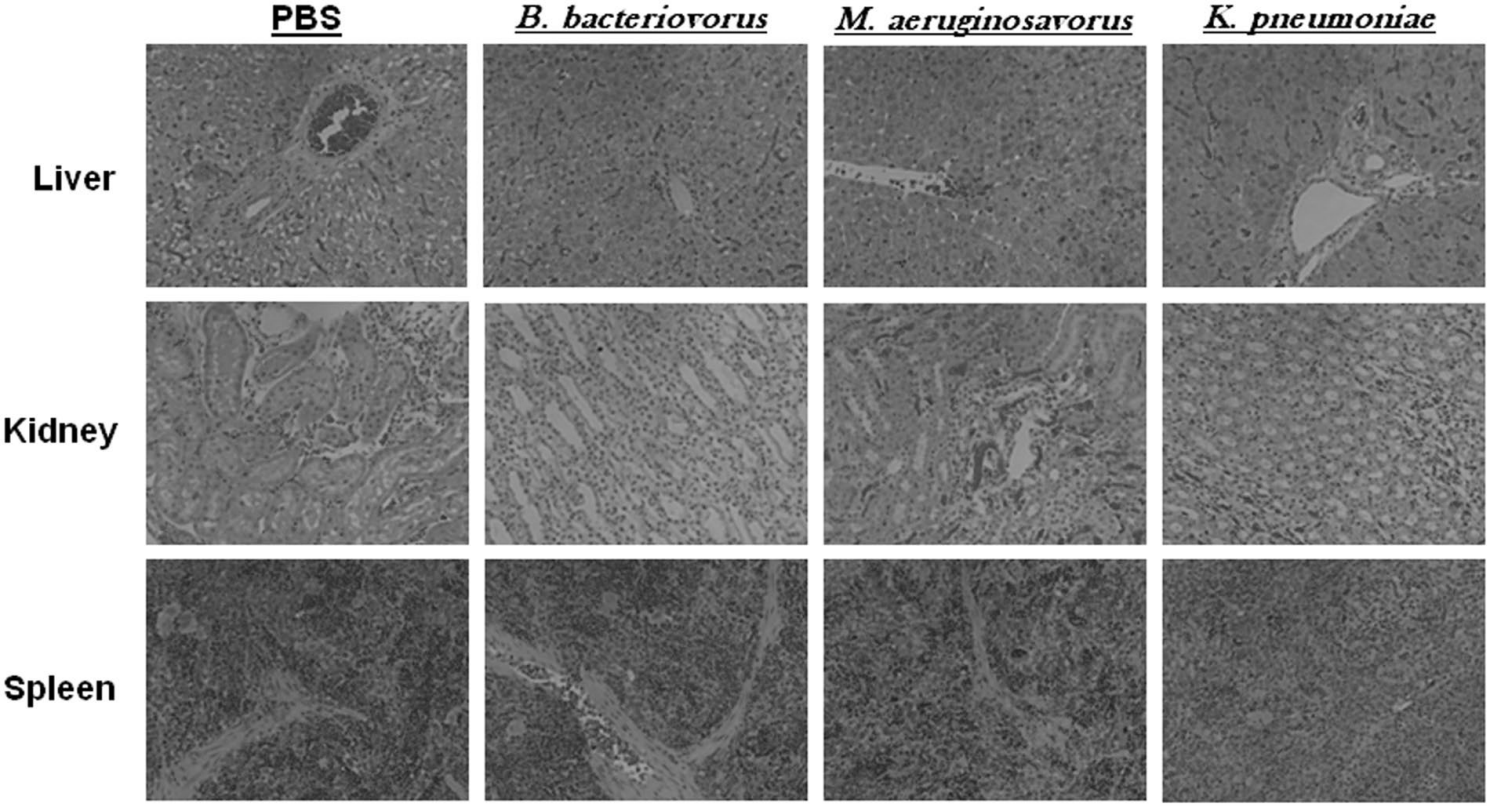
Histological examination of rat organs after intravenous inoculation of predatory bacteria. SD rats were intravenously inoculated (through tail vein injection) with PBS, *B. bacteriovorus*, *M. aeruginosavorus*, or *K. pneumoniae*. Histological examination of harvested rat livers, kidneys, and spleens exposed to *B. bacteriovorus* and *M. aeruginosavorus* revealed no abnormal pathology compared to rats treated with PBS. All images are representative micrographs that were taken at 18 hours post-inoculation and at X40 total magnification.

Once again, all rats that were inoculated with predatory bacteria were found to be visually healthy (Table 1). One *K. pneumoniae*-treated rat succumbed to infection at 18 hours post-inoculation (Table 1). Histological examination of liver and kidney tissue from rats inoculated with either *B. bacteriovorus* 109J or *M. aeruginosavorus* exhibited no noticeable differences compared to control (PBS) at any time point examined (Fig. 1). Extramedullary hematopoiesis was observed in spleen samples harvested from all groups. In contrast, the spleens of rats treated with *K. pneumoniae* showed prominent individual cell death within the periarteriolar lymphoid sheath at 18 hours post-injection (Fig. 1). In addition, one *K. pneumoniae*-treated rat exhibited obvious individual cell death within the red and white pulp at 18 hours post-inoculation. Collectively, and in direct comparison with *K. pneumoniae* sepsis, the data suggest that introducing predatory bacteria directly into the blood has no visually apparent effect on rat morbidity or histopathology.

### Host immune response

**Figure 2:**
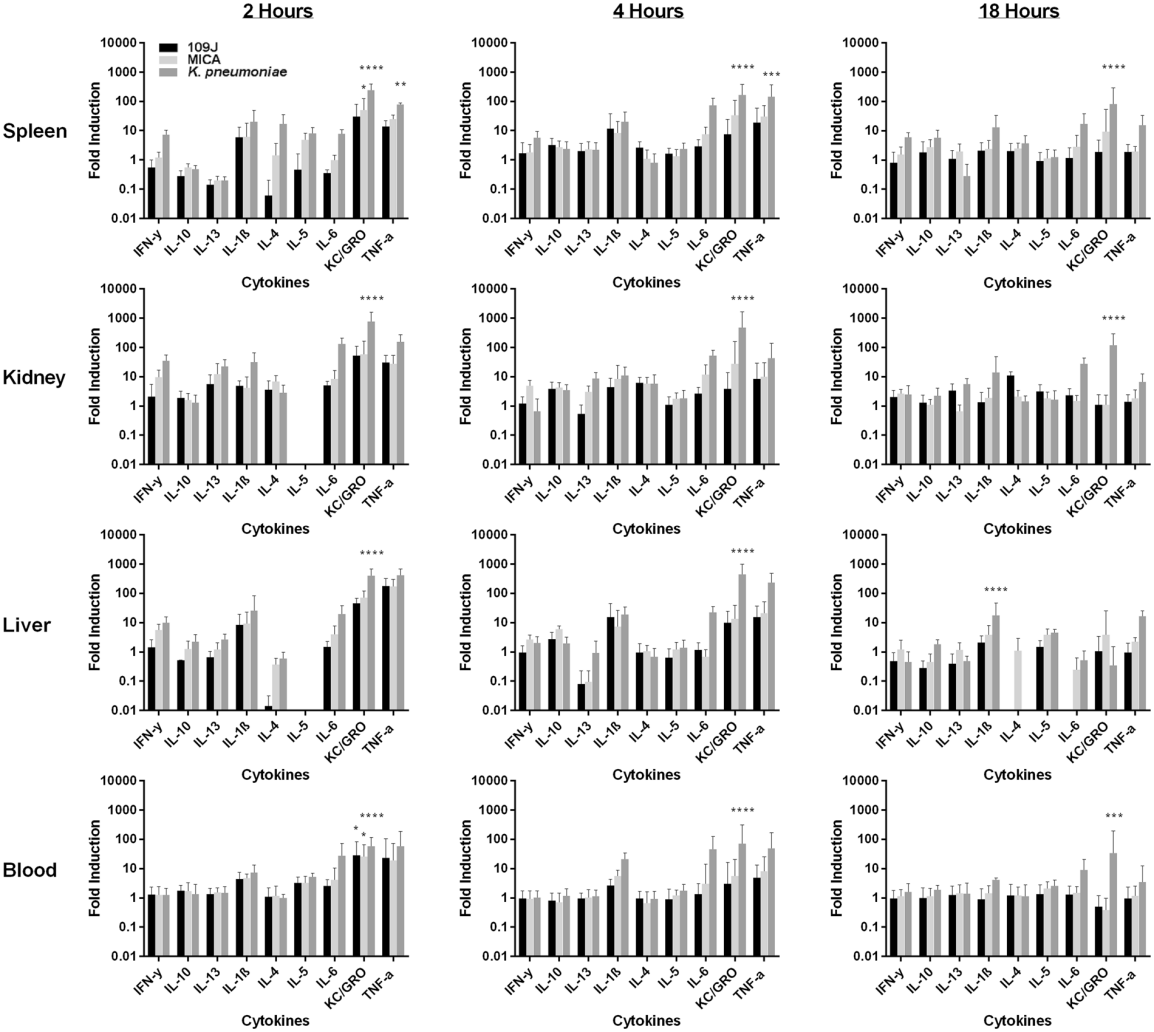
Inflammatory protein profile within rat blood and organs in response to intravenous inoculation of predatory bacteria. ELISA analysis of IL-1β, IL-4, IL-5, IL-6, IL-10, IL-13, CXCL-1/KC, IFNγ, and TNF in response to tail vein injection with predatory bacteria relative to PBS control was performed. Rats were injected with PBS, *B. bacteriovorus* 109J, or *M. aeruginosavorus* (and also *K. pneumoniae* [Kp] as a control). Inflammatory proteins were assessed within the spleen, liver, kidney and blood at two, four, and 18 hours post-inoculation. Twelve rats per treatment group (seven for *K. pneumoniae*) were used at each time point. Data are combined from two independent experiments. Data represent means ± standard errors of the means. Significant differences between treatment groups and respective control were determined using ANOVA (*P < 0.05; **P < 0.01; ***P < 0.001; ****P < 0.0001).

To determine the host inflammatory response to intravenous inoculation of predatory bacteria, enzyme linked-immunosorbent assays (ELISA) were performed on blood, kidney, liver, and spleen samples harvested from the aforementioned short term experiment. We detected 28.6- and 23.3-fold increases of KC/GRO and TNFα, respectively, at two hours post-injection in the blood of rats injected with *B. bacteriovorus* 109J; 25.3- and 18.7-fold increases, respectively, were detected in the blood of *M. aeruginosavorus*-treated rats (Fig. 2). In the blood of *K. pneumoniae*-treated rats, 7.2-, 5.3-, 27.7-, 57.2-, and 57.2-fold increases of IL-1β, IL-5, IL-6, KC/GRO, and TNFα, respectively, were detected (Fig. 2). At four hours post-injection, 5.5-, 5.5-, and 8.2-fold increases of IL-1β, KC/GRO, and TNFα, respectively, were still detected in *M. aeruginosavorus*-treated blood (Fig. 2). However, by 18 hours post-injection, all assayed inflammatory cytokines and chemokines in the blood of rats treated with either predatory bacterial species returned back to baseline levels. In contrast, we detected 9.1- and 33.8-fold increases in IL-6 and KC/GRO, respectively, in the blood of *K. pneumoniae*-treated rats at 18 hours post-inoculation (Fig. 2).

In the spleen, we detected 6.0-, 30.6-, and 14.1-fold increases of IL-1β, KC/GRO, and TNFα in rats injected with *B. bacteriovorus* 109J at two hours post-inoculation; 6.0-, 50.1-, and 25.0-fold increases, respectively, were detected in *M. aeruginosavorus*-treated rats (Fig. 2). *K. pneumoniae*-treated spleens demonstrated 7.3-, 20.0-, 17.1-, 8.1-, 7.7-, 243.5-, and 76.9-fold increases of IFNγ, IL-1β, IL-4, IL-5, IL-6, KC/GRO, and TNFα, respectively (Fig. 2). By 18 hours post-inoculation, inflammatory cytokines and chemokines detected within spleens treated with predatory bacteria returned back to baseline levels, with the exception of KC/GRO which still exhibited a 9.4-fold increase in *M. aeruginosavorus*-treated spleens (Fig. 2). In stark contrast, we still detected 6.0-, 5.8-, 13.0-, 17.3-, 80.8-, 15.4-fold increases of IFNγ, IL-10, IL-1β, IL-6, KC/GRO, and TNFα, respectively, in the spleens of *K. pneumoniae*-treated rats at 18 hours post-inoculation (Fig. 2).

In the liver, 8.2-, 44.7-, and 176.7-fold increases of IL-1β, KC/GRO, and TNFα, respectively, was detected in *B. bacteriovorus* 109J-treated rats at two hours post-inoculation; 5.5-, 9.2-, 71.2-, and 172.1-fold increases of IFNγ, IL-1β, KC/GRO, and TNFα, respectively, were detected in *M. aeruginosavorus*-treated rats (Fig. 2). In *K. pneumoniae*-treated livers, 9.8-, 25.4-, 19.7-, 406.3-, and 410.7-fold increases of IFNγ, IL-1β, IL-6, KC/GRO, and TNFα, respectively, were detected (Fig. 2). Once again, inflammatory cytokines and chemokines in rats treated with predatory bacteria returned to baseline levels by 18 hours post-inoculation. The livers of *K. pneumoniae*-treated rats still exhibited 17.8- and 16.7-fold increases of IL-1β and TNFα, respectively, at 18 hours post-injection (Fig. 2).

In the kidney, 5.5-, 5.0-, 5.0-, 52.3-, and 30.8-fold increases of IL-13, IL-1β, IL-6, KC/GRO, and TNFα, respectively, was detected in *B. bacteriovorus* 109J-treated rats at two hours post-inoculation; 9.8-, 12.1-, 6.8-, 8.3-, 57.6-, and 27.5-fold increases of IFNγ, IL-13, IL-4, IL-6, KC/GRO, and TNFα, respectively, were detected in *M. aeruginosavorus*-treated rats (Fig. 2). In *K. pneumoniae*-treated kidneys, 34.6-, 22.3-, 31.7-, 129.2-, 754.1-, and 155.4-fold increases of IFNγ, IL-13, IL-1β, IL-6, KC/GRO, and TNFα, respectively, were detected (Fig. 2). Once again, all tested inflammatory cytokines and chemokines returned to baseline levels by 18 hours post-inoculation. In comparison, we still detected 5.5-, 14.1-, 27.4-, 118.3-, and 6.6-fold increases of IL-13, IL-1β, IL-6, KC/GRO, and TNFα, respectively, in the kidneys from *K. pneumoniae*-treated rats at 18 hours post-inoculation (Fig. 2).

**Figure 3:**
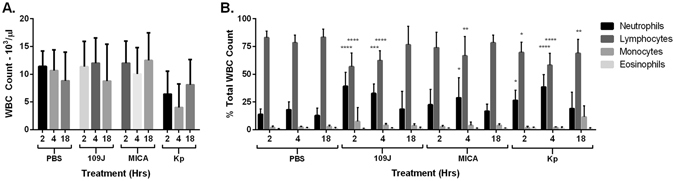
Inflammatory cell response to intravenous injection of predatory bacteria. In order to profile the (**A**) total white blood cell counts and (**B**) inflammatory cell response in the blood due to predatory bacteria, rats were injected through the tail vein with PBS, *B. bacteriovorus* 109J, *M. aeruginosavorus*, or *K. pneumoniae* (Kp). Blood samples were assessed at two, four, and 18 hours post-injection. Data represent means ± standard errors of the means. Significant differences between treatment groups and respective PBS control were determined using ANOVA.

To examine the effect of intravenous injection of predatory bacteria on the host immune cell blood profile, 100 µl of blood from each rat was harvested at two, four and 18 hours post-injection, when white blood cell (WBC) counts were performed and cell types present determined. We observed no difference in total WBC counts in the blood of rats injected with either predatory bacteria compared to control, while a decrease in WBC counts was detected in the blood of *K. pneumoniae*-treated rats at every time point examined (Fig. 3A). A 2.8- and 3.7-fold increase in the percentage of neutrophils and monocytes, respectively, (accompanied with a 0.68-fold decrease in the percentage of lymphocytes) circulating in the blood was detected in the blood of *B. bacteriovorus* 109J-treated rats at two hours post-injection (Fig. 3B). However, levels of individual WBCs returned to baseline levels by 18 hour post-inoculation. No substantial differences in the levels of individual immune cell types were observed between *M. aeruginosavorus*-treated rats and control at any time point (Fig. 3B). In contrast, a 2.0- and 3.0-fold increase in the percentage of neutrophils and eosinophils, respectively, was detected in the blood of *K. pneumoniae*-treated rats at two hours post-inoculation (Fig. 3B). At four hours post-inoculation, a 2.1-fold increase in the percentage of neutrophils in the blood was observed, while a 4.1-fold increase in the percentage of monocytes was still detected at 18 hours post-injection in rats treated with *K. pneumoniae* (Fig. 3B). Taken altogether, the data suggest that injecting predatory bacteria directly into the blood does not provoke a substantial and sustained immune response in rats.

### Predatory bacterial dissemination

**Figure 4:**
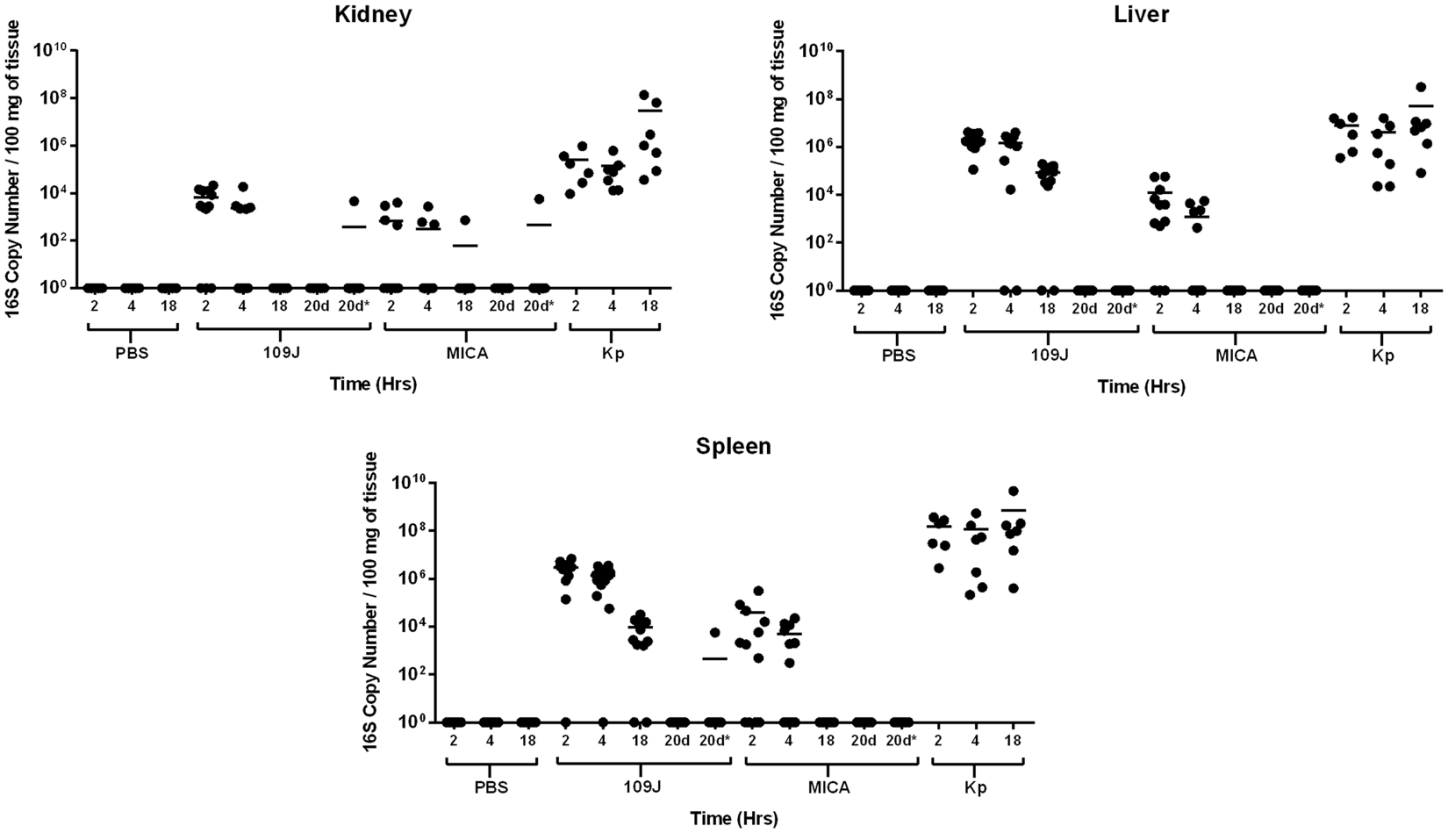
Predatory bacterial dissemination within host. qPCR detection of predatory bacteria within the host was performed. The kidneys, livers, and spleens were probed for *B. bacteriovorus* 109J, or *M. aeruginosavorus* (MICA), and *K. pneumoniae* at two, four, and 18 hours post-injection. Twelve rats per treatment group (seven for *K. pneumoniae*) were analyzed at each time point. Each data point represents a single rat’s respective bacterial load. Horizontal lines represent the mean of the results from each treatment set. Data are combined from the results of two independent experiments.

To determine the predatory bacterial load disseminated to various organs after intravenous inoculation of predatory bacteria, quantitative polymerase chain reaction (qPCR) was performed on the harvested kidney, liver, and spleen samples. In all organs examined, levels of predatory bacteria 16S rRNA decreased over time. At two hours post-injection, *B. bacteriovorus* 109J was detected in the kidneys of 9/12 rats (at levels ranging from 2.2 × 10^3^ to 2.1 × 10^4^ copy numbers) and *M. aeruginosavorus* in 4/12 rats (4.5 × 10^2^ to 4.0 × 10^3^) (Fig. 4). By 18 hours post-inoculation, *M. aeruginosavorus* was detected in the kidneys of only 1/12 rats (7.3 × 10^2^), while no detectable *B. bacteriovorus* 109J was observed in any of the twelve rats (Fig. 4). In comparison, *K. pneumoniae* was detected in the kidneys of all twelve rats sacrificed at two, four and 18 hours post-injection (Fig. 4).

Similar results were observed between liver and spleen samples. In the liver, *B. bacteriovorus* 109J was detected in all twelve rats (1.1 × 10^5^ to 4.2 × 10^6^) and *M. aeruginosavorus* in 9/12 rats (5.0 × 10^2^ to 5.8 × 10^4^) at two hours post-inoculation (Fig. 4). In the spleen, *B. bacteriovorus* 109J was detected in 11/12 rats (1.4 × 10^5^ to 6.9 × 10^6^) and *M. aeruginosavorus* in 8/12 rats (4.9 × 10^2^ to 3.1 × 10^5^) at two hours post-injection (Fig. 4). At 18 hours, *B. bacteriovorus* 109J was still detected in the livers of 10/12 rats (2.4 × 10^4^ to 2.0 × 10^5^), while no detectable *M. aeruginosavorus* was observed in the livers of any of the twelve rats (Fig. 4). Comparably, *B. bacteriovorus* 109J was also detected in the spleens of 10/12 rats (1.6 × 10^3^ to 3.2 × 10^4^), while, again, no detectable *M. aeruginosavorus* was observed in the spleens of any of the twelve rats at 18 hours post-injection (Fig. 4). In stark contrast, high levels (>10^6^) of *K. pneumoniae* were observed in every rat at every time point examined (Fig. 4).

Kidney, liver and spleen samples harvested from the initial long-term and multiple bacteremia experiments (see “Host morbidity and histopathology”) were also probed for the presence of predatory bacteria 16 S rRNA. No *B. bacteriovorus* 109 J or *M. aeruginosavorus* was detected in any of the organs probed at 20 days after a single injection of predatory bacteria (Fig. 4). In the multiple bacteremia event model, *B. bacteriovorus* 109J was detected in the kidney of only 1/12 rats (4.6 × 10^3^) and the spleen of 1/12 rats (5.7 × 10^3^) at 20 days post-initial injection; *M. aeruginosavorus* was detected in the kidney (5.7 × 10^3^) of 1/12 rats (Fig. 4). No predatory bacteria were detected in the livers in any of the long-term rat models (Fig. 4). In conclusion, the data indicate that predatory bacteria injected into the blood stream and disseminated to other organs are quickly and efficiently cleared by the host.

### Pathogen inoculation and treatment

To determine the ability of predatory bacteria to attenuate bacterial burden introduced directly into the blood stream, we injected 2.3 × 10^8^ CFU/rat of *K. pneumoniae* into the tail veins of twelve rats (‘experimental group’), while twelve more rats were injected with the vehicle, PBS (‘control group’). Four rats from each group were treated with PBS, 2.3 × 10^8^ PFU/rat of *B. bacteriovorus* 109J, or 1.3 × 10^8^ PFU/rat of *M. aeruginosavorus* at 30 minutes, six, 12, and 18 hours post-infection. Rats were sacrificed at 24 hours post-infection when blood, liver, kidney, and spleen samples were harvested. The blood and homogenized organs were then plated on MacConkey agar to assess for *K. pneumoniae* load.

**Figure 5:**
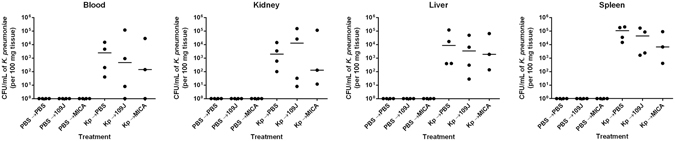
*K*. *pneumoniae* bacterial burden within rat blood and organs after treatment scheme #1 with predatory bacteria. *K*. *pneumoniae* (or PBS for control groups) was initially introduced into rats via intravenous inoculation. Animals were then treated via tail vein injection with PBS, *B. bacteriovorus* 109J or *M. aeruginosavorus* (MICA) at 30 minutes, 6, 12, and 18 hours post-injection. At 24 hours, blood was collected and kidneys, livers, and spleens were harvested, homogenized, and plated on MacConkey agar plates to recover *K. pneumoniae* CFUs. Four rats per treatment group were used at each time point. Each data point represents a single rat’s respective bacterial load. Horizontal lines represent the median of the results from each treatment set. Significant differences between treatment groups and respective control were performed using the Mann-Whitney test.

At 24 hours, all animals infected with *K. pneumoniae* and treated with PBS were found to be unresponsive and with hunched postures, while the majority of animals treated with predatory bacteria were responsive and visually healthy. One animal infected with *K. pneumoniae* and treated with *M. aeruginosavorus* did succumb to infection at approximately 21 hours post-infection. Within the ‘experimental group,’ we recovered a median of 2.4 × 10^3^ CFU/mL of *K. pneumoniae* in the blood, 8.6 × 10^3^ CFU/mL in the liver, 2.0 × 10^3^ CFU/mL in the kidney, and 1.1 × 10^5^ CFU/mL in the spleen in rats initially infected with *K. pneumoniae* and treated with PBS (Fig. 5). In rats treated with *B. bacteriovorus* 109J, we recovered a median of 4.6 × 10^2^ CFU/mL of *K. pneumoniae* in the blood, 3.4 × 10^3^ CFU/mL in the liver, 1.3 × 10^4^ CFU/mL in the kidney, and 4.4 × 10^4^ CFU/mL in the spleen (Fig. 5). In rats treated with *M. aeruginosavorus*, we isolated a median of 1.4 × 10^2^ CFU/mL of *K. pneumoniae* in the blood, 1.9 × 10^3^ CFU/mL in the liver, 1.3 × 10^2^ CFU/mL in the kidney, and 6.7 × 10^3^ CFU/mL in the spleen (Fig. 5). No *K. pneumoniae* was isolated from the blood or any organ harvested from rats in the ‘control group’ (Fig. 5). The results suggest that predatory bacteria were not able to reduce *K. pneumoniae* burden in a rat model of bacteremia.

In a second attempt, we included a second strain of *B. bacteriovorus* (strain HD100) and modified the treatment schedule of predatory bacteria to 30 minutes, five, 9.5, and 14 hours (every ~4.5 hours) post-*K. pneumoniae* infection. Sixteen rats were infected with 2.3 × 10^8^ CFU/rat of *K. pneumoniae* (‘experimental group’), while sixteen more were injected with PBS (‘control group’). Four rats from each group were treated with PBS, 3.0 × 10^8^ PFU/rat of *B. bacteriovorus* 109J, 3.8 × 10^8^ PFU/rat of *B. bacteriovorus* HD100, or 3.3 × 10^7^ PFU/rat of *M. aeruginosavorus* at the respective time points. Rats were sacrificed at 16 hours post-infection when blood, liver, kidney, and spleen samples were harvested and assessed for *K. pneumoniae* load.

**Figure 6:**
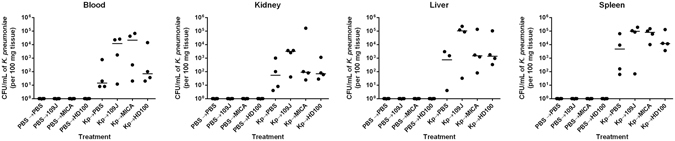
*K. pneumoniae* bacterial burden within rat blood and organs after treatment scheme #2 with predatory bacteria. *K. pneumoniae* (or PBS for control groups) was initially introduced into rats via intravenous inoculation. Animals were then treated via tail vein injection with PBS, *B. bacteriovorus* 109J, HD100, or *M. aeruginosavorus* (MICA) at 30 minutes, 5, 9.5, and 14 hours post-injection. At 16 hours, blood was collected and kidneys, livers, and spleens were harvested, homogenized, and plated on MacConkey agar plates to recover *K. pneumoniae* CFUs. Four rats per treatment group were used at each time point. Each data point represents a single rat’s respective bacterial load. Horizontal lines represent the median of the results from each treatment set. Significant differences between treatment groups and respective control were performed using the Mann-Whitney test.

Once again, no *K. pneumoniae* was isolated from the blood or any organ harvested from rats in the ‘control group’ (Fig. 6). Within the ‘experimental group,’ we recovered a median of 1.4 × 10^1^ CFU/mL of *K. pneumoniae* in the blood, 7.5 × 10^2^ CFU/mL in the liver, 5.4 × 10^1^ CFU/mL in the kidney, and 4.7 × 10^3^ CFU/mL in the spleen in rats treated with PBS (Fig. 6). In rats treated with *B. bacteriovorus* 109J, we recovered a median of 1.2 × 10^4^ CFU/mL of *K. pneumoniae* in the blood, 1.0 × 10^5^ CFU/mL in the liver, 3.1 × 10^3^ CFU/mL in the kidney, and 9.6 × 10^4^ CFU/mL in the spleen (Fig. 6). In rats treated with *B. bacteriovorus* HD100, a median of 6.8 × 10^1^ CFU/mL of *K. pneumoniae* in the blood, 1.4 × 10^3^ CFU/mL in the liver, 7.0 × 10^1^ CFU/mL in the kidney, and 1.2 × 10^4^ CFU/mL in the spleen were recovered (Fig. 6). In rats treated with *M. aeruginosavorus*, we isolated a median of 2.1 × 10^4^ CFU/mL of *K. pneumoniae* in the blood, 1.5 × 10^3^ CFU/mL in the liver, 9.0 × 10^1^ CFU/mL in the kidney, and 8.0 × 10^4^ CFU/mL in the spleen (Fig. 6). Collectively, the data indicate that intravenous administration of predatory bacteria is unable to reduce numbers of *K. pneumoniae* in a mammalian bacteremia model.

## Discussion

In response to the alarming rise of antibiotic-resistant infections, scientists have begun to explore new ways to treat bacterial infections; one of these approaches is the use of predatory bacteria^15^. Recently, we demonstrated the ability of predatory bacteria to attenuate bacterial burden *in vivo* within the lungs of rats^19^. In this study, we extend this approach to determine if predatory bacteria can reduce bacterial burden introduced directly into the rat vasculature.

We began by assessing the safety of administering predatory bacteria via tail vein injection in SD rats. PBS, *B. bacteriovorus* 109J, *M. aeruginosavorus*, or *K. pneumoniae* were injected directly into the bloodstream and rats were monitored for up to 20 days. The two predatory bacterial genera were used in order to determine any differences between periplasmic (*B. bacteriovorus*) and epibiotic (*M. aeruginosavorus*) predation. *K. pneumoniae* was used as the pathogen because we sought to use a clinically-relevant strain and it has been used as the Gram-negative pathogen of choice in all other studies done by our group^16, 19^. Bloodstream infections caused by carbapenem-resistant *K. pneumoniae* strains have been on-the-rise and are known to have high mortality rates^21, 22^. In addition, a group of rats were re-injected at 10 days post-initial injection to model a multiple bacteremia event. All rats that were administered predatory bacteria via single or multiple injections exhibited no signs of morbidity. Furthermore, histological examination of liver and kidney tissue revealed no adverse histopathology due to predatory bacteria compared to control (PBS-treated). In comparison, tissue from rats infected with *K. pneumoniae* exhibited histological abnormalities, while one rat succumbed to infection at 18 hours post-infection. Extramedullary hematopoiesis (EMH) was observed in the spleens of rats from all treatment groups, including PBS. EMH refers to the production of leukocytes outside the bone marrow^23^. In humans, both the liver and spleen are capable of this activity; however, EMH is typically associated with only acute demand^24^.

A previous study performed by our laboratory examining the effect of intravenous inoculation of predatory bacteria in C57BL/6 mice reported similar results^16^. In that study, a group of mice were injected with *B. bacteriovorus* 109J and observed for up to 20 days; another group of mice were re-injected with *B. bacteriovorus* 109J at 10 days post-initial injection to model a repeat exposure. All mice injected with *B. bacteriovorus* 109J were found to be healthy and, combined with the results of our current study, confirms that intravenous injection of predatory bacteria is nontoxic and safe to administer to murine mammals.

We next determined the host inflammatory response to intravenous injection of predatory bacteria. We observed an increase of TNFα and KC/GRO in the blood due to predatory bacteria at two hours post-inoculation; however, this response was not sustained and levels of inflammatory cytokines returned to baseline levels by 18 hours. Inflammatory cytokines and chemokines in the spleen, liver, and kidney due to predatory bacteria also followed a similar pattern as that of in the blood, increasing at two hours post-injection and returning to baseline by 18 hours. In stark contrast, most cytokines assessed were still highly elevated at 18 hours post-inoculation in rats treated with *K. pneumoniae*. We also observed an increase in the percentage of neutrophils and monocytes circulating in the blood after predatory bacteria administration. Furthermore, dissemination analysis revealed that predatory bacteria did not efficiently disperse to and remain in other organs after tail vein injection. In all organs examined levels of predatory bacteria 16S rRNA decreased over time, with complete clearance in most rats by 20 days post-injection.

The observed inflammatory responses are signatures of activation of the primary innate immune response. TNFα is produced by many immune cell types, including neutrophils, and is an important first-response regulator of inflammation^25^. KC/GRO is secreted by neutrophils and macrophages, and is known to attract neutrophils in a positive feedback loop^25^. Thus, the increase in TNFα and KC/GRO seen early after initial exposure to predatory bacteria correlates well with the increase in neutrophils and monocytes measured in the blood. The previous study assessing intravenous inoculation of predatory bacteria in mice reported very similar results. Mice exhibited a 53-fold increase in KC/GRO accompanied with a 3.5- and 4.7-fold increase in the percentage of neutrophils and monocytes present in the blood after injection with predatory bacteria^16^. Furthermore, another study demonstrated *B. bacteriovorus* is also cleared from the hindbrains of zebrafish larvae by neutrophils and macrophages^20^. Therefore, it is most likely that predatory bacteria are being cleared from the blood by neutrophils or other innate immune cells.

The lack of a strong and sustained inflammatory response to predatory bacteria compared to that seen in response to *K. pneumoniae* may be explained by the presence of an altered lipopolysaccharide (LPS). The classical negatively-charged LPS expressed on the surface of Gram-negative bacteria are pathogen-associated molecular patterns that activate innate immune responses to protect the host from infection^26^. *B. bacteriovorus* expresses a neutral-charged LPS which was demonstrated to be weakly immunogenic *in vitro* ^27^. Future work will determine if *M. aeruginosavorus* contains an altered LPS, as well.

To determine whether predatory bacteria could attenuate bacterial burden administered directly into the bloodstream, we injected *K. pneumoniae* into the tail veins of rats and followed with four treatments of predatory bacteria at 30 minutes, six, 12, and 18 hours post-infection to model an antibiotic dosing regimen. Rats were sacrificed at 24 hours post-infection when blood, liver, kidney, and spleen samples were harvested, homogenized, and plated on MacConkey agar, a medium selective for Gram-negative and enteric bacteria, such as *K. pneumoniae*. Rats infected with just *K. pneumoniae* were not responsive and exhibited abnormal postures, while rats treated with predatory bacteria appeared visually healthy. However, we did not observe significant reduction in *K. pneumoniae* concentrations in the blood or any of the organs assessed due to *B. bacteriovorus* 109 J or *M. aeruginosavorus* treatment.

Due to the results we obtained, we hypothesized that the time between each predatory bacteria treatment (six hours) may have been too long and allowed *K. pneumoniae* concentrations to rebound, possibly explaining the lack of morbidity seen in rats infected with *K. pneumoniae* and treated with predatory bacteria. For this reason, we performed a second trial treating rats infected with *K. pneumoniae* with predatory bacteria every 4.5 hours, with a shortened total experiment length of 16 hours. We included an additional strain of *B. bacteriovorus* (strain HD100) in the second attempt, in order to determine if results were strain specific. However, we again did not observe significant reduction of *K. pneumoniae* in the blood, liver, kidney, or spleen.

The fact that predatory bacteria were able to reduce pathogen burden in the lungs, but not the blood, of rats may signal that predatory bacteria (as currently administered) may be a more viable treatment when topically applied to or targeted to a more contained site of infection. The lack of effect in the blood could be explained as simply as the predators’ inability to locate their prey when injected directly into the vasculature. Alternatively, as we observed an increase in proinflammatory cytokines, chemokines, and innate immune cells in the blood due to predatory bacteria (and *K. pneumoniae*) already by two hours post-inoculation, host immune response elements recruited to the blood may also be clearing out the predators before they can prey efficiently on the pathogen. We hypothesize that predatory bacteria therapy may be useful for infections occurring in immune-privileged sites, such as urinary tract infections, due to the lack of immune response elements and therefore the ability of the predators to potentially persist longer at the site of infection.

The results signal that predatory bacteria may not be able to attenuate pathogen burden associated with a blood infection. It is possible that the dosing schemes we employed are not optimal for treating blood infections with predatory bacteria. Nonetheless, this study does provide further support that predatory bacteria are safe and nontoxic to administer in mammals. Future studies will attempt different dosing regimens, as well as explore the feasibility to treat infections occurring at immune-privileged sites, to continue to determine if predatory bacteria are a viable treatment for bacterial infections.

## Methods

### Bacteria, strains, and growth conditions

The predatory bacteria used for this study were *Bdellovibrio bacteriovorus* 109J (ATCC 43826), *B. bacteriovorus* HD100^28^ (ATCC 15356) and *Micavibrio aeruginosavorus* strain ARL-13^7^. *Klebsiella pneumoniae* (ATCC 43816) was used as the pathogen and grown in Luria-Bertani (LB) medium. Predatory bacteria were cultured and processed as previously described^19^. *Escherichia coli* (WM3064) was grown overnight in LB medium supplemented with 0.3 mM DAP to be used as prey. Predator stock-lysates were prepared by co-culturing the predators with host cells in HEPES buffer (25 mM) supplemented with 3 mM MgCl_2_ and 2 mM CaCl_2_. The co-cultures were incubated at 30 °C until the culture cleared (stock-lysates). To develop high concentrations of *Bdellovibrio* for inoculation experiments, 10 mL of a washed overnight culture of *E. coli* (WM3064) cells (~1 × 10^9^ CFU/ml) was re-suspended in 80 mL of HEPES medium containing 10 mL of predatory bacteria from stock lysates and incubated on a rotary shaker at 30 °C for 24 h. Similarly, 25 mL of *Micavibrio* stock lysates were added to 25 mL of *E.coli* in 200 mL of HEPES media and incubated for 72 hours to get a concentrated *Micavibrio* culture. Once the co-cultures appeared clear, they were passed twice through a 0.45-μm-pore-size Millex filter (Millipore) to remove any prey and cell debris (filtered lysate). Filtered lysates were pelleted three times by centrifugation at 29,000 g for 45 min using a Sorvall LYNX 4000 centrifuge (Thermo Fisher Scientific Inc.) to further purify and concentrate predator samples. Each time, the pellet was washed and re-suspended in 50 ml of phosphate-buffered saline (PBS). For the final wash, the predator pellet was re-suspended in 1 to 2 ml of PBS solution to reach final optical densities at 600 nm (OD_600_) of 0.2 ± 0.02 for *B. bacteriovorus* and 0.1 ± 0.02 for *M. aeruginosavorus*, which corresponded to PFU values of between ~1.0 × 10^9^ and 1.0 × 10^10^ PFU/ml and between ~1.0 × 10^8^ and 1.0 × 10^9^ PFU/ml, respectively. Predator cell concentrations were quantified using the standard double-layered agar method^29^. Fifty µl of the predator samples was plated on DAP-supplemented LB agar and tryptic soy broth (TSB)-blood plates to verify that the samples had no contaminants or prey cells. Since the predatory bacteria were used directly after isolation, the actual viable predator dose was known only a few days after each experiment, as the PFUs appeared. Therefore, in some experiments, mainly involving *M. aeruginosavorus*, the inoculation sizes differed somewhat. The actual predator inoculation doses are specified for each experiment.

### Rats

Wild-type male Sprague Dawley rats (4 to 6 weeks old) were purchased from Charles River Laboratories (Wilmington, MA). All rats were housed under pathogen-free conditions at the Rutgers New Jersey Medical School animal facility. All experiments were performed in accordance with the protocols approved by the Institutional Animal Care and Use Committee of Rutgers New Jersey Medical School (protocol #15012) and the Animal Care and Use Review Office of the US Army Medical Research and Material Command were followed in handling the animals.

### Intravenous inoculation

Predatory bacteria were introduced by intravenous inoculation through the tail vein to model a systemic blood infection. Animals were anesthetized with 4% isoflurane-oxygen for 5 min. using an isoflurane vaporizer. Two hundred and fifty μl of purified bacterial suspension was injected into the tail vain using a 26 G polyurethane catheter (2619PUR; Covidien). Rats were inoculated with PBS, *B. bacteriovorus* 109J, *M. aeruginosavorus* ARL-13, or *K. pneumoniae*. Animals were separated in cages according to treatment group and time point to be sacrificed to assure no cross contamination. Animals were visually evaluated for signs of illness or discomfort throughout the experiment. Liver, kidney, spleen, and blood samples were collected at two, four, and 18 hrs post-inoculation for use in histological examinations, inflammatory protein analysis, and bacterial dissemination experiments.

### Inflammatory protein analysis (ELISA)

Liver, kidney, and spleen samples were harvested in Lysing Matrix D tubes (MP Biomedicals) containing 1.0 mL of PBS with protease inhibitor. A FastPrep-24 instrument (MP Biomedicals) was used to homogenize samples at 5.0 m/s for 60 s before storing them at −80 °C. At time of analysis, samples were thawed and centrifuged at >13,000 × g relative centrifugal force (RCF) for 10 min at 4 °C. Resulting supernatant was filtered through a 0.22 μm-pore-size filter at 12 × g RCF for 4 min. Cytokines were measured using a V-Plex proinflammatory Panel 2 (rat) kit (K15059D-1; Meso Scale Discovery) according to manufacturer’s instructions, and read on a SECTOR imager 2400 (Meso Scale Discovery).

### Nucleic acid extraction

Samples were prepared as previously described^19^. Liver, kidney, and spleen samples designated for RNA extraction were harvested in Lysing Matrix D tubes containing 1.0 mL of TRIzol (Invitrogen). A FastPrep-24 instrument was used to homogenize samples at 5.0 m/s for 60 s before storing them at −80 °C. Total RNA was extracted as previously described. Samples were thawed and then centrifuged at >13,000 × g RCF for 20 min. at 4 °C. Two hundred μl of chloroform was added to the transferred supernatant. The reaction was centrifuged at >13,000 × g RCF for 15 min. at 4 °C. The aqueous layer was transferred and an equal volume of isopropanol was added to it. The reaction was centrifuged at >13,000 × g RCF for 15 min. to pellet the precipitated RNA. The isopropanol was removed and the RNA pellets remaining were washed twice with 500 μl of ice-cold 70% ethanol. The samples were then resuspended in 30 μl of nuclease-free water. The “RNA Cleanup” protocol in the RNeasy Mini Kit (Qiagen) was used to purify the RNA. The purified total RNA was then stored at −80 °C.

### Bacterial dissemination

Harvested organs were stored in TRIzol to preserve RNA for future dissemination analysis. Extracted total RNA underwent cDNA synthesis using iScript Reverse Transcription Supermix (Bio-Rad Laboratories) according to manufacturer’s instructions. Primers specifically targeting the 16S rRNA gene of each predatory bacterial strain were synthesized according to ref. 19. qPCR was performed on the samples in triplicate, with each reaction mixture consisting of the following components: template (1.0 μl of cDNA synthesized as described above), SsoAdvanced Universal SYBR green Supermix (Bio-Rad Laboratories), and a 500 nM (for 109J and *Micavibrio*) or 900 nM (for HD100) concentration of each primer (synthesized at the Rutgers New Jersey Medical School Molecular Resource Facility). A CFX384 Touch Real-Time PCR Detection System (Bio-Rad Laboratories) was used with the following protocol: 50 °C for 2 min (1 cycle), 95 °C for 10 min (1 cycle), 95 °C for 15 s and 60 °C for 1 min (40 cycles), and 95 °C for 15 s, 60 °C for 15 s, and 95 °C for 15 s (1 cycle). For each qPCR run, a 10-fold dilution series of the standard (purified DNA from each predatory strain) was assessed in triplicate to validate qPCR performance and facilitate quantification (E = 103.6%, R^2^ = 0.994, Slope = −3.239). Negative controls (no template) were included as well in each qPCR run. 16S rRNA copy numbers were calculated using “Calculator for determining the number of copies of a template” (URI Genomics and Sequencing Center; http://cels.uri.edu/gsc/cndna.html)^30^.

### Blood profiling

At two, four, and 18 hours post-injection, 100 μl of blood samples were collected from rats via heart puncture. Samples were sent to ANTECH Diagnostics (New Hyde Park, NY, USA) for blood cell profiling.

### Histological examination

Liver, kidney, and spleen samples were collected and stored in formalin at 4 °C. All histological samples were examined by a pathologist blind to the treatment group of the specimen. Formalin-fixed organ segments from infected rats were paraffin embedded and stained with hematoxylin and eosin (H&E) for analysis of cellular composition as previously described. An EVOS FL Cell Imaging System (Life Technologies, Carlsbad, CA) was used to photograph and analyze the stained sections.

### Pathogen inoculation and treatment

Animals were anesthetized and inoculated intravenously with 250 μl of *K. pneumoniae* as previously described. In the first trial, 250 μl of predatory bacteria were inoculated at 30 min, six, 12, and 18 hours post-*K. pneumoniae* infection. Rats were then euthanized at 24 hours post-inoculation when blood, liver, kidney, and spleen samples were harvested for downstream analysis. Organ samples designated for CFU were placed on ice in Lysing Matrix D tubes containing 1.0 mL of PBS. Samples were immediately homogenized at 6.0 m/s for 1 min on a FastPrep-24 instrument. Homogenized samples were serially diluted and plated on MacConkey agar to determine *K. pneumoniae* concentrations. In the second trial, rats were inoculated with 250 μl of predatory bacteria at 30 min, 4.5, 9, and 13.5 hours post-*K. pneumoniae* infection. Animals were then euthanized at 16 hours post-inoculation.

### Statistical Analysis

ELISA data are presented as means ± standard errors of the means; significant differences between the data from the treated samples and the data from the respective controls were determined using analysis of variance (ANOVA). *K. pneumoniae* reduction data are presented as medians; the Mann-Whitney test was used in analysis of significant differences between treatment groups and controls. A *P* value of 0.05 was considered significant. All statistical analyses and graphs were prepared in GraphPad Prism 6.05.
